# Integrated Exon Level Expression Analysis of Driver Genes Explain Their Role in Colorectal Cancer

**DOI:** 10.1371/journal.pone.0110134

**Published:** 2014-10-21

**Authors:** Mohammad Azhar Aziz, Sathish Periyasamy, Zeyad Al Yousef, Ibrahim AlAbdulkarim, Majed Al Otaibi, Abdulaziz Alfahed, Glowi Alasiri

**Affiliations:** 1 Medical Genomics, King Abdullah International Medical Research Center, Riyadh, Saudi Arabia; 2 Bioinformatics, King Abdullah International Medical Research Center, Riyadh, Saudi Arabia; 3 Surgery, National Guard Health Affairs, Riyadh, Saudi Arabia; University of Bonn, Institut of experimental hematology and transfusion medicine, Germany

## Abstract

Integrated analysis of genomic and transcriptomic level changes holds promise for a better understanding of colorectal cancer (CRC) biology. There is a pertinent need to explain the functional effect of genome level changes by integrating the information at the transcript level. Using high resolution cytogenetics array, we had earlier identified driver genes by ‘Genomic Identification of Significant Targets In Cancer (GISTIC)’ analysis of paired tumour-normal samples from colorectal cancer patients. In this study, we analyze these driver genes at three levels using exon array data – gene, exon and network. Gene level analysis revealed a small subset to experience differential expression. These results were reinforced by carrying out separate differential expression analyses (SAM and LIMMA). ATP8B1 was found to be the novel gene associated with CRC that shows changes at cytogenetic, gene and exon levels. Splice index of 29 exons corresponding to 13 genes was found to be significantly altered in tumour samples. Driver genes were used to construct regulatory networks for tumour and normal groups. There were rearrangements in transcription factor genes suggesting the presence of regulatory switching. The regulatory pattern of AHR gene was found to have the most significant alteration. Our results integrate data with focus on driver genes resulting in highly enriched novel molecules that need further studies to establish their role in CRC.

## Introduction

There is a wealth of information at omics level that associates cytogenetics and gene expression changes leading to colorectal cancer (CRC). The integration of gene expression and copy number (CN) data to identify DNA CN alterations that induce changes in the expression levels of the associated genes is a common task in cancer studies [1]–[3]. The central dogma of molecular biology has thus been addressed at two important levels. There have been many reports providing evidence of changes at the genome level in the form of copy number aberration [4], single nucleotide polymorphisms, loss of heterozygosity that attempt to understand the molecular events associated with colorectal cancer. These somatic or hereditary changes have different mechanism of contributing to initiation and progression of CRC. Loss and gain of crucial chromosomal regions leading to deletion or amplification of cancer related genes has been very well established. The functional significance of these molecular events has been measured using different tools and algorithms. Genes targeted by somatic copy-number alterations (SCNAs), in particular, play central roles in oncogenesis and cancer therapy [5]. Several tools have been made available to assess the potential of genes that get affected by SCNAs in causing colorectal cancer. 'Genomic Identification of Significant Targets in Cancer' (GISTIC) tool has successfully been used in identifying 'driver SCNAs' based on the frequency and amplitude of observed events [6], [7]. The second aspect of changes happening in tumour cells is at the transcription level. Differential expression analysis has been carried out to find out important genes playing a role in causing colorectal cancer. There could be several mechanisms by which the SCNA affected genes exert their effect at functional level. Amplifications and deletions in the genomic region are reflected in the transcript levels and could be detected by carrying out expression microarray based studies. By employing exon arrays, we gain extra dimension of the events happening at the exon level, which may lead to alternative splicing resulting in different gene isoforms. Alternative splicing is a crucial step in the generation of protein diversity and its misregulation is observed in many human cancer types [8].

The quest to explore the relationship between copy number changes and the expression level of affected genes/exons has received limited success owing to a number of reasons [9]. Technological improvements in the array design for cytogenetics as well as transcriptomics have improved the accuracy and precision of the data generated. Combining this with better analytical techniques and algorithms, possibilities of finding target genes responsible for causing colorectal cancer has further increased.

Past few decades have seen a quest for finding novel genes that can serve as therapeutic targets or biomarkers. However, genes or proteins do not function alone but interact with each other to form networks or pathways so as to carry out biological functions [10]. Network-based approaches to finding biomarkers more closely represent *in vivo* molecular biology where a perturbation in one gene may affect many downstream genes. Cancer has thus been rightly addressed as a systems biology disease [11] as opposed to diseases caused by changes in few genes or mutations. Reconstructing gene regulatory networks in healthy and diseased tissues is therefore critical to understanding cancer phenotypes and devising effective therapeutics [12].

With the availability of tools and techniques to capture the molecular changes happening at different stages of the central dogma with increased precision and accuracy, we are yet to develop an integrated comprehensive picture that could help us in finding better targets for colorectal cancer. In the present study, we aim to integrate the information from the cytogenetic analysis with the exon level expression data using paired normal-tumour samples from colorectal cancer patients. A set of driver genes suggested by GISTIC analysis (termed as ‘driver genes’ from now onwards) were queried at gene, exon and network level. Both DNA and RNA for cytogenetics and transcriptomic studies, respectively, were extracted from the same tissue in a single workflow to minimize variation. This study provides evidence for explaining different possible mechanisms by which SCNA affected driver genes can exert their functional effect. A subset of driver genes were found to show gene level changes in expression. Most of these genes also indicated the exon level changes resulting in the formation of different isoforms. Network of GISTIC genes showed a clear shift in the transcription factors (TF) regulation. ATP8B1 gene was found to have novel association with colorectal cancer at cytogenetic, gene and exon level.

## Materials and Methods

The study is approved by the ethics committee and Institutional Review Board (IRB) of King Abdullah International medical Research Center after due review process of the ethical aspects of the proposal. The necessary procedural and ethical consent forms were signed by each patient prior to sample collection.

### Sample collection and RNA extraction

Sample collection was done as described previously [13]. The type and stage of all patient samples are provided in Table S1. The study was approved by institutional review board after the due process. Patients were consented and the records maintained in an approved manner. RNA was extracted from the same piece of tissue that was used to extract DNA for cytogenetic studies in a single workflow. Maceherey Nagel trio prep kit (Germany) was used to extract DNA and RNA in the same protocol. Quality and quantity was checked using Nanodrop (Thermo Fisher Scientific, USA).

### Exon microarray

GeneChip Human Exon 1.0 ST Arrays along with WT Terminal Labelling and Controls Kit and Hybridization, Wash, and Stain Kit were obtained from Affymetrix USA. Ambion WT Expression Kit was obtained by Ambion, USA. 31 Tumour and 29 normal samples from 32 patients were processed. The data was extracted using Expression Console software from Affymetrix, USA. Quality control was carried out using Principal Component Analysis (PCA) and Integromics biomarker suite (TIBCO spotfire). All the data is deposited in GEO database with an accession number GSE50421.

### Data Analysis

Before carrying out any gene/exon level analysis, principal component analysis (PCA) was done to identify outliers. Data from 4 normal samples and 7 tumour samples was subsequently removed.

### Data analysis specifically with driver genes

#### Gene level analysis

For checking expression levels of 144 driver gene list generated by GISTIC analysis (13), we employed two different softwares – Expression console and AltAnalyze [14]. Two different softwares were used to confirm our results using independent methods. Signal estimates were derived from the CEL files of 60 samples (29 normal and 31 tumour) using Robust Multi-Array Average (RMA) for normalizing the data. The core exon-level probe sets were used to summarize the gene expression levels.

The same list of 144 driver genes with expression values calculated using ‘Altanalyze’ was used for inference based (GENIE3) pathway/network analysis.

#### Exon level expression analysis

Altanalyze program was used to evaluate alternative splicing in driver genes. The raw data was filtered to remove probe sets that were considered to be non-expressed. A splicing score for filtered exons was calculated using splicing index method and exon/intron/splicing annotation were assigned to these results. A splice index p-value cut-off of <0.05 was used to filter alternative exon results. AltExonViewer – a component of Altanalyze and DomainGraph - a Cytoscape plugin were used to visualize splice index values and alternatively spliced exons. The splicing index (SI) value was calculated as described in [15]. Briefly, SI is log_2_ ratio of normalized intensities of tumour and normal samples. In our analysis ‘sample 1’ in the numerator was normal and ‘sample 2’ in the denominator was tumour.

#### Causal network analysis

For network analysis, knowledge and inference based approaches were used. For inference based approaches, GENIE3 [16], [17] was used to generate gene regulatory networks for tumour and normal samples. To generate the network, driver genes were classified as TF genes and target genes. Although 1000 interactions were inferred for each of the group, an interaction score>0.1 was chosen as a cut-off value. Using this information the regulatory networks were independently inferred for tumour and normal samples using Cytoscape.

### Data analysis with entire probeset

#### Integromics Biomarker Discovery Suite

In order to find differentially expressed genes in our dataset, without any prior bias, data from CEL files was analyzed using the Integromics software (TIBCO Spotfire, USA) pipeline for affymetrix exon 1.0 ST arrays. Quantile normalization was done after removing the outliers using PCA. Both Significance Analysis of Microarrays (SAM) and Linear Models for MicroArray data (LIMMA) analyses was carried out with a cut-off of 0.01 for adjusted p-value and fold change of>1 or <−1. Two different yet complimentary methodologies were used to make our results more confident. Gene ontology enrichment was carried out on a list of 760 differentially expressed genes obtained from LIMMA analysis.

#### Ingenuity pathway Analysis

List of 760 genes from SAM/LIMMA analysis done using Integromics was used to perform ‘core’ and ‘biomarker’ analyses. Core analysis was done as described before [13]. For biomarker analysis following filters were used: Consider only molecules where (species  =  Human) AND (tissues/cell lines  =  KM-12 OR HCT-116 OR RKO OR Colon Cancer Cell Lines not otherwise specified OR COLO205 OR HT29 OR HCC-2998 OR HCT-15 OR SW-480 OR Other Colon Cancer Cell Lines OR Tissues and Primary Cells not otherwise specified OR SW-620) AND (diseases  =  Cancer) AND ((biomarker applications  =  All Biomarker Applications) AND (biomarker diseases  =  colon cancer OR colon carcinoma OR colon neoplasm OR colorectal adenoma OR colorectal cancer OR colorectal carcinoma)).

### Results

**Figure 1:**
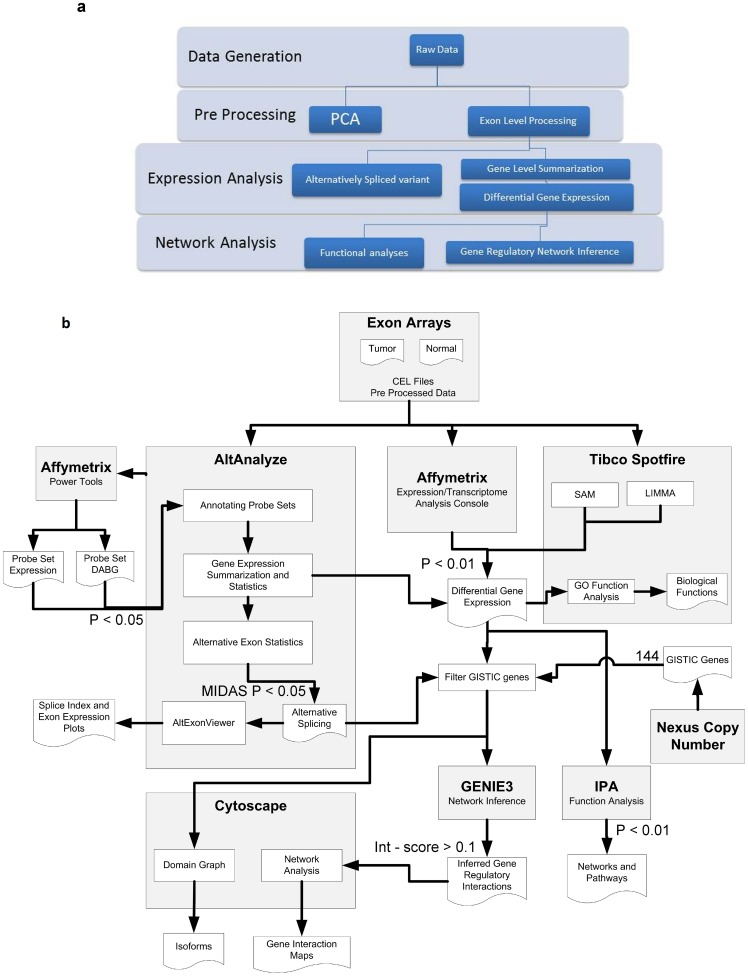
Flow diagram for Analysis Strategy. A)The entire analyses is categorized into fours stages from ‘Data Generation’ to ‘Network Analyses'. B) Analysis strategy using different programs is displayed in this diagram. There are three components of the analysis – Gene, Exon and Network handled by different programs. Gene level analyses are conducted using ‘Affymetrix, Expression/Transcriptome analysis console’ and ‘Tibco Spotfire’. Exon level analysis is carried out by ‘AltAnalyze’ and ‘Affymetrix power tools’. Network analyses employed ‘GENIE3’, ‘IPA’ and ‘Cytoscape’. ‘Nexus Copy Number’ is a program used in earlier studies to eventually generate a list of 144 driver genes.

The analysis strategy leading to following results is illustrated in Figure 1a&b.

#### A small subset of genes identified by GISTIC show a significant change in expression level

**Figure 2:**
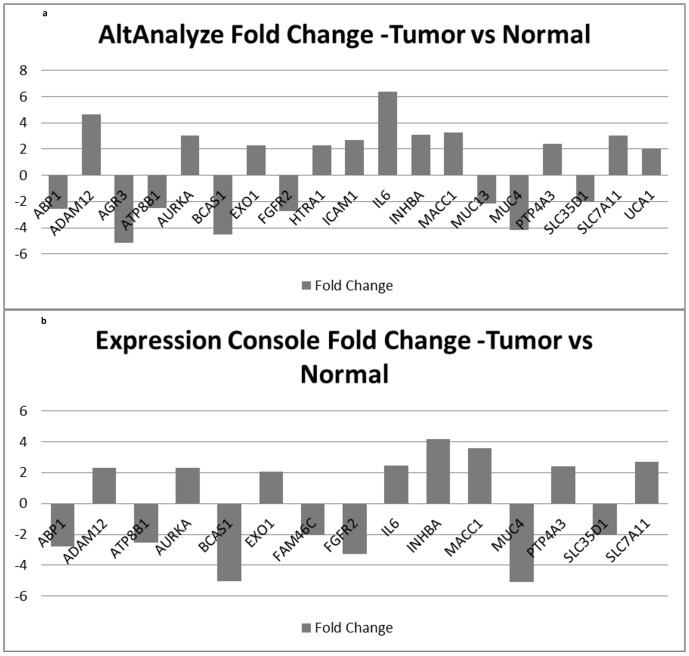
Significant change in expression value at gene level was observed in 20/144 genes. Two different algorithms were used to measure expression values from Exon array data to support the results. AltAnalyze (1a) and Expression Console (1b) show complimentary results with maximum changes observed in BCAS1, INHBA, IL6 and MUC4 genes.

We studied expression patterns of driver genes at gene level using AltAnalyze and Expression Console softwares. These analyses produced complimentary results and yielded a list of 20 genes that were found to have a significant fold change of greater than 2 and a p-value <0.01 [Table 1]. 9 genes experienced a down regulation. BCAS1 with highest GISTIC score of 5.323 was among the most significantly downregulated genes. 11 genes showed an upregulation with IL6 and INHBA showing the highest fold change [Figure 2a (AltAnalyze), 2b (Expression Console)]. Fold change values of all 144 driver genes are given in Table S2.

**Table 1:** Driver Genes from GISTIC analysis showing more than two fold change in expression value as calculated by two different programs – AltAnalyze and Expression Console.

Gene Symbol	GISTIC score	AltAnalyze	Expression Console
BCAS1^a^^,^ b	5.323	−4.50771	−5.02
AURKA^a^^,^ b	4.772	3.044088	2.31
ATP8B1^a^	2.326	−2.49709	−2.55
IL6^a^^,^ b	2	6.353318	2.45
INHBA^a^^,^ c	1.944	3.071911	4.18
PTP4A3^a^^,^ b ^,^ c	1.892	2.366951	2.41
ABP1^a^^,^ b	1.859	−2.54991	−2.78
AGR3	1.772	−5.12325	N/A
MACC1	1.772	3.272925	3.57
UCA1	1.155	2.058481	N/A
FGFR2^a^^,^ b	0.892	−2.72455	−3.28
ADAM12^a^^,^ b	0.871	4.646741	2.33
MUC13	0.704	−2.15979	N/A
MUC4^a^^,^ b ^,^ c	0.66	−4.18466	−5.06
SLC7A11^a^	0.637	3.051363	2.72
ICAM1	0.532	2.655218	N/A
EXO1	0.405	2.268313	2.05
FAM46C^a^	0.271	−1.7528	−2.03
HTRA1	0.231	2.301062	N/A
SLC35D1^a^	0.217	−2.0238	−2.06

N/A  =  No annotation for these genes were found in the analysis program.

a Genes found to be differentially expressed in SAM/LIMMA analyses. CLDN7 and LOX genes were additional driver genes that were differentially expressed.

b Genes found to have high splice index values.

c Genes found to be eligible as biomarkers for colorectal cancer.

Three significantly down regulated genes (BCAS1, ABP1 and AGR3) were found in amplified regions of the genome whereas upregulated HTRA1 was found mostly in regions of loss. These genes experienced a change in their transcription factor in tumour and normal samples [Table 2].

**Table 2:** Differentially regulated genes found to have incoherent expression levels and genomic changes.

Gene	Genomic level	Gene level (AA, EC)	Network level
ABP1	Gain 53.33, Loss 13.33	−2.55, −2.78	TF in Normal = CTCFL TF Tumour = Unknown
AGR3	Gain 60, Loss 0	−5.12	TF in Normal = SMAD2 TF Tumour = Unknown
BCAS1	Gain 80, Loss 0	−4.5, −5	TF in Normal = SMAD4 TF Tumour = Unknown
HTRA1	Gain 6.7, Loss 26.7	2.3	TF in Normal = CTCFLTF Tumour = AHR

AA  =  Fold change value as calculated by AltAnalyze program.

EC  =  Fold change value as calculated by Expression Console program.

TF  =  Transcription Factor. Unknown is the TF that is not found in the driver genes.

**Figure 3:**
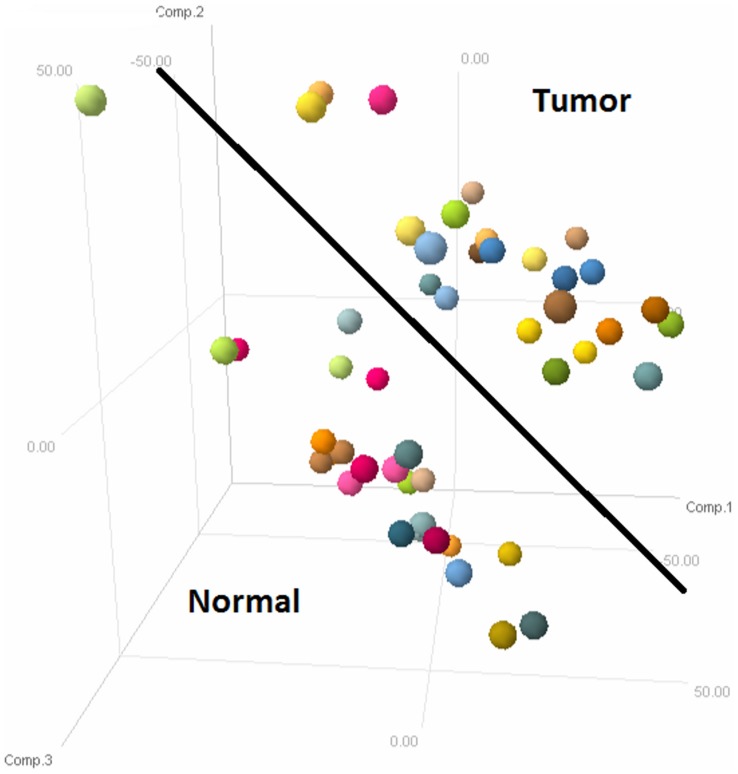
Principal Component Analysis of Exon array data from 32 patients. 60 samples from 32 patients were subjected to PCA and the outliers were removed. 4 normal and 7 tumour samples were removed from the final analysis.

**Figure 4:**
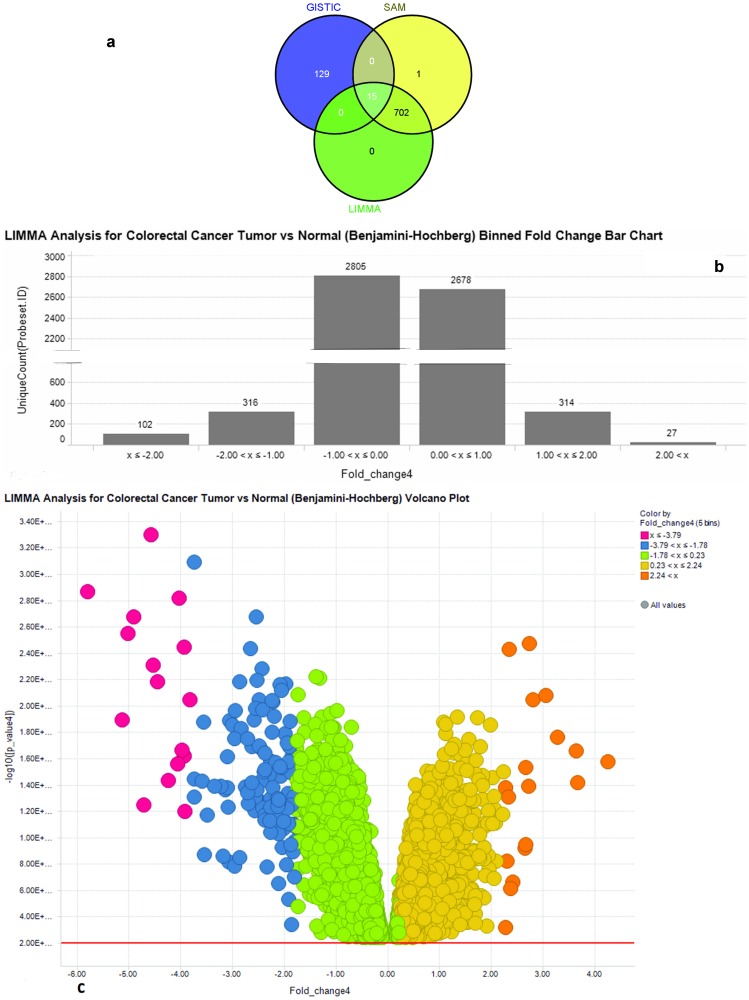
Differential Expression analysis of genes yielded genes common with GISTIC analysis. (a) Venn diagram of common genes among GISTIC, SAM and LIMMA analyses. All genes were annotated and compared using IPA ‘compare’ function. 43 genes from Integromics analyses were not mapped by IPA.15 genes are common among all three analyses. (b) Binned fold change bar chart of LIMMA analysis. Total 6242 genes were having adjusted p-value <0.01 of which 759 showed significant fold change (<−1 or>1). (c) Volcano plot showing highly significant genes (pink  = downregulated, orange = upregulated) in terms of p- value and fold change.

Differential expression analysis using tumour-normal paired sample data of exon arrays from 32 patients was carried out. After removing outliers using PCA [Figure 3], 25 normal and 24 tumour samples were found suitable for further analyses. We employed non-parametric (SAM) and parametric (LIMMA) methods and found complimentary results. 6242 genes were differentially expressed with adjusted p-value of <0.01. 760 genes were found to be differentially regulated (fold change>1or <−1) of which 15 genes were common with driver genes from GISTIC analysis [Figure 4a-c and Figure S1]. BCAS1, AURKA, ATP8B1, IL6 and INHBA were the differentially regulated genes found to be among the top scorers in list of driver genes.

#### Significant changes in isoform expression is exhibited by genes identified by GISTIC analysis

**Figure 5:**
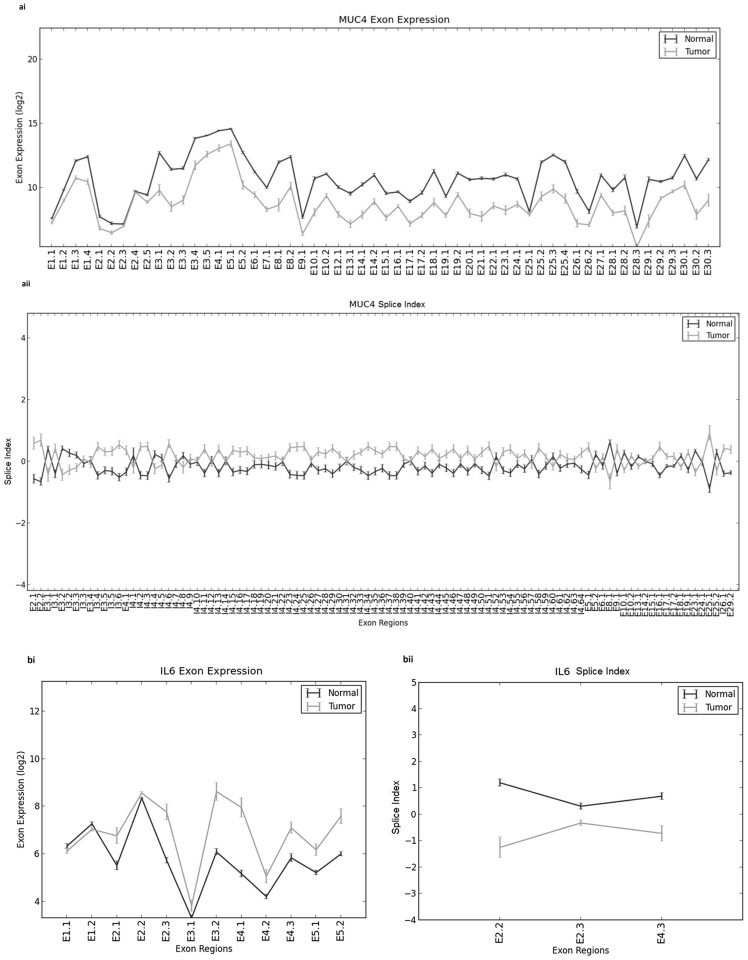
Differential Expression at exon level was observed in thirteen GISTIC genes. Exon expression (i) and splice index (ii) values were mapped for both tumour and normal samples for twenty nine exons affecting thirteen genes. Exon 25-1 of MUC4 gene (a) shows highest negative splice index value (a ii) whereas exon 2-2 of IL6 (b) showed highest value of 2.44 (b ii). Exon expression and splice index values for rest 27 exons are provided as supplementary figure 1.

Exon level analysis of 144 driver genes was carried out. 29 exons belonging to 13 genes were shown to have significant changes in isoform expression as reflected in their splice index scores [Table 3]. While exons E25-1 of MUC4 and E3-2 of PTP4A3 showed high negative SI values, exons E2-2 of IL6 and E21-2 of ADAM12 showed high positive SI values. Negative SI values indicate exons are enriched in tumour samples and are skipped or repressed in normal samples and vice versa for positive SI values. For MUC4 gene, exons E25-1, E2-2, E2-1, I4-6 and I3-6 recorded negative SI values and exon E8-1 recorded a positive SI value. Exons E2-2 and E4-3 of IL6 recorded positive SI values. [Figure 5 ai-ii & bi-ii and figure S2]. Exons in MYLK and ANK3 showed significant change in splicing pattern but did not show a significant fold change in gene expression value. Microarray Detection of Alternative Splicing (MiDAS) p-value range of 0.01–0.04 was observed in the filtered results.

**Table 3:** Significant changes in exon level expression of driver genes.

Probeset	GeneID	Exon ID	Regulation Call	SI	SI p-value	MiDAS p-value
2712254	MUC4	E25-1	Upregulated	−1.80873	2.79E-08	0.021609
3118824	PTP4A3	E3-2	Upregulated	−1.61711	9.95E-12	0.012046
2712381	MUC4	E2-2	Upregulated	−1.3477	7.19E-07	0.026458
3310161	FGFR2	E11-1	Upregulated	−1.29699	4.55E-08	0.023093
3310118	FGFR2	E29-1	Upregulated	−1.27723	1.86E-09	0.01652
3910391	BCAS1	E4-3	Upregulated	−1.25187	3.35E-07	0.021612
3031671	ABP1	E11-1	Upregulated	−1.24918	1.22E-10	0.014292
3310159	FGFR2	E12-1	Upregulated	−1.17574	2.50E-07	0.026414
2712382	MUC4	E2-1	Upregulated	−1.15152	1.33E-05	0.037457
3290897	ANK3	E60-1	Upregulated	−1.14825	1.19E-04	0.041986
2712354	MUC4	I4-6	Upregulated	−1.12743	3.39E-07	0.028327
2712361	MUC4	I3-6	Upregulated	−1.05374	8.25E-07	0.03115
3290925	ANK3	E53-5	Upregulated	−1.05177	2.60E-05	0.040567
3268341	HTRA1	E1-8	Downregulated	1.007325	5.53E-10	0.016611
3310147	FGFR2	E17-1	Downregulated	1.086154	2.78E-06	0.031111
3268338	HTRA1	E1-5	Downregulated	1.086203	3.38E-11	0.013645
2388253	EXO1	E18-1	Downregulated	1.08806	3.11E-05	0.044937
3311901	ADAM12	E10-1	Downregulated	1.089706	1.70E-09	0.018986
3910807	AURKA	E1-2	Downregulated	1.105387	2.78E-07	0.026314
3820445	ICAM1	E3-3	Downregulated	1.222886	3.84E-07	0.027735
2712282	MUC4	E8-1	Downregulated	1.267574	1.63E-05	0.039593
2692532	MYLK	E13-1	Downregulated	1.273764	4.27E-07	0.030277
3268334	HTRA1	E1-1	Downregulated	1.288045	1.63E-12	0.011119
3310142	FGFR2	E20-1	Downregulated	1.403702	1.07E-09	0.015743
3268335	HTRA1	E1-2	Downregulated	1.404729	1.43E-12	0.011016
2992599	IL6	E4-3	Downregulated	1.406659	4.60E-05	0.044146
3311894	ADAM12	E11-1	Downregulated	1.420516	1.99E-10	0.016023
3311853	ADAM12	E21-2	Downregulated	2.016734	1.96E-09	0.018149
2992593	IL6	E2-2	Downregulated	2.448861	1.85E-07	0.027456

List of 29 exons corresponding to 13 genes are listed along with their respective splice index and MiDAS p values.

Probeset IDs are according to Affymetrix database

SI  =  Splice Index

MiDAS  =  Microarray Detection of Alternative Splicing

#### Causal Network analysis displays switch in transcription factors in tumor samples

**Figure 6:**
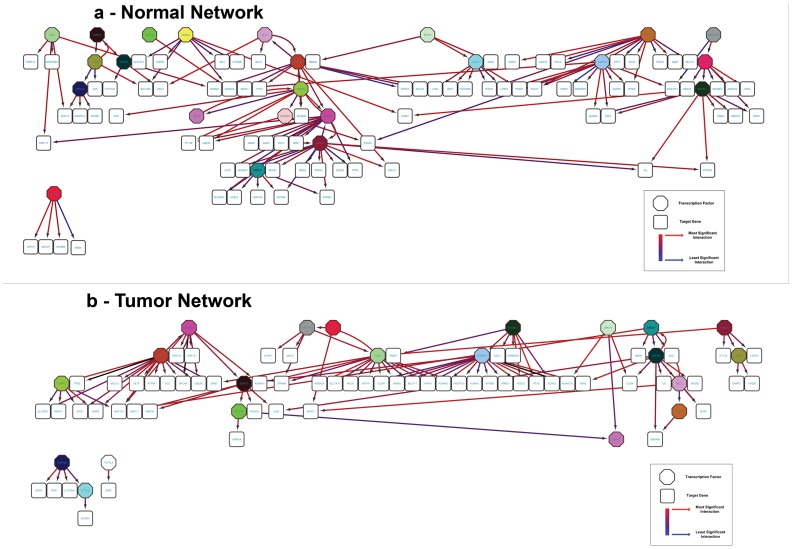
Gene Regulatory Network Inference diagram for Tumor and Normal Groups. A hierarchical network topology is used to visualize the degrees of interaction between transcription factor genes and target genes. (a) The inferred network for tumour group showing RUVBL1 as master regulator. (b) The inferred network for normal group showing TSHZ1as master regulator.

Causal Network analysis displays switch in transcription factors in tumour samples. The most significant influence of TF genes in tumour and normal samples was reflected in the alteration in number of directed edges [Table 4]. CHAF1A, AHR, PRPF4B, ZNF200, SMAD2, RUVBL1, SMAD4, TSHZ1, CTCFL and CEBPE were among the top influencers. The regulatory hierarchy between these groups changed with respect to TFs. While RUVBL1 transformed into a master regulator in tumours, TSHZ1 has lost its capacity to master regulate other genes in tumours [Figure 6a]. Significant rearrangement in modularity is also observed between these two groups. More target genes function as modules in tumours as observed in modules regulated by AHR, CHAF1A and PRPF4B. Further, there is significant regulatory crosstalk among the genes in the normal group [Figure 6b]. While the tumours have lost the scale-free properties in its regulatory interaction, normal group exhibit approximate scale-free out degree distributions, signifying the potential of TF to regulate host of target genes. The tumours do not show this type of regulation which indicate unidirectional feed forward regulatory mode.

**Table 4:** Transcription factor genes showing significant change in their effect as represented by the change in number of outbound and inbound edges in tumour and normal samples.

Transcription Factor	In bound edges (Normal, Tumour)	Out bound edges (Normal, Tumour)
***CHAF1A***	***1,1***	***16,12***
***AHR***	***0,2***	***5,14***
***PRPF4B***	***2,1***	***11,14***
***ZNF200***	***1,2***	***3,9***
***SMAD2***	***3,1***	***13,8***
***RUVBL1***	***1,0***	***8,7***
***CEBPE***	***2,1***	***7,5***
SMAD7	1,2	2,5
HIST1H1B	0,1	2,4
MLL3	1,1	3,4
***SMAD4***	***2,0***	***14,4***
ZNF217	1,1	6,4
ZNF442	1,0	3,4
SPO11	0,0	3,3
NHLH2	0,1	1,2
TP53	1,1	4,2
***CTCFL***	***1,1***	***6,1***
SMAD3	0,0	4,1
TCF7L2	1,0	6,1
***TSHZ1***	***0,1***	***12,1***

#### Functional analysis of differentially expressed genes confirms their role in colorectal cancer and reveal important pathways and biomarkers

**Figure 7:**
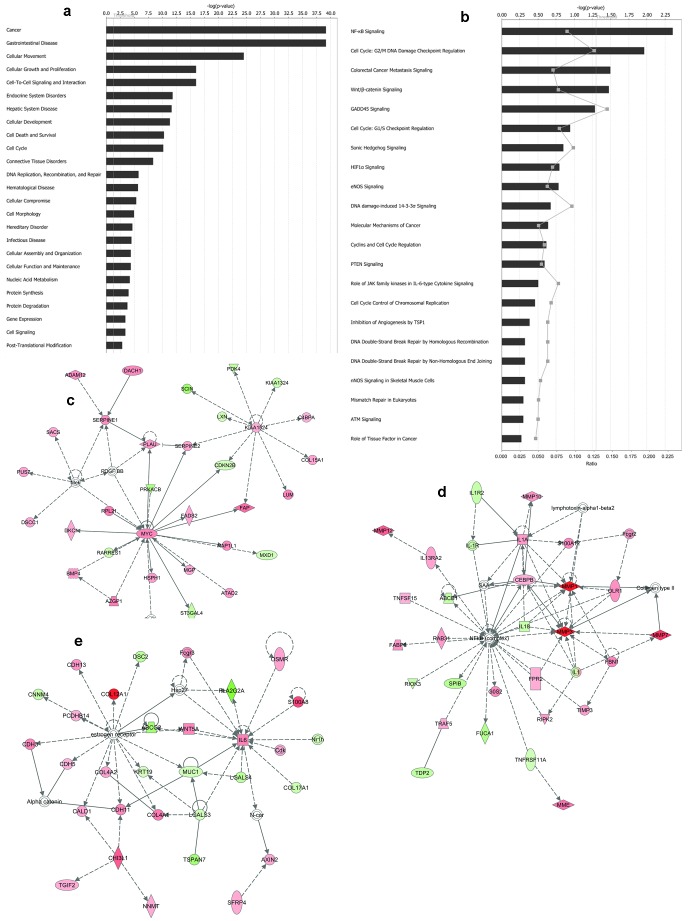
Core Analysis of Differentially Expressed Genes using IPA. Core analysis using IPA was carried out using set of 760 genes that were differentially expressed in tumour samples. Important biological functions (a) pathways (b) and networks (c-e) were revealed by this analysis.

Gene Ontology enrichment of 760 differentially expressed genes obtained from SAM/LIMMA analyses showed cell division, mitosis and cell adhesion to be the most significant biological processes affected [figure S3]. Ingenuity pathway analysis of 760 genes showed cancer and gastrointestinal disease among the top functions followed by cellular movement growth and proliferation [Figure 7a]. NF-kB signalling, cell cycle G_2_/M DNA damage checkpoint regulation, colorectal cancer metastasis were among the top scoring pathways followed by agranulocyte adhesion [figure 7b]. TGFB1 was among the top upstream regulators. Network analysis suggests MYC, MMP and IL6 genes as important nodes [Fig 7c-e]. Biomarker analysis reveals 28 molecules relevant for colorectal cancer [Table 5]. INHBA, CLDN7 and MUC4 were eligible as biomarkers and are common with GISTIC, SAM and LIMMA analyses results.

**Table 5:** Biomarker molecules among the differentially expressed genes.

Symbol	Fold Change	p-value	Location	Type(s)
ANGPT2	2.055	6.89E-14	Extracellular Space	growth factor
CD34	1.104	4.27E-10	Plasma Membrane	other
CD44	1.256	9.28E-14	Plasma Membrane	enzyme
CDH13	1.338	4.13E-11	Plasma Membrane	other
CLDN7	−1.030	1.81E-09	Plasma Membrane	other
CXCL10	1.073	8.50E-03	Extracellular Space	cytokine
EREG	1.352	2.39E-05	Extracellular Space	growth factor
GDF15	1.191	6.33E-06	Extracellular Space	growth factor
IGF2	1.398	3.85E-04	Extracellular Space	growth factor
IL8	3.641	2.55E-17	Extracellular Space	cytokine
INHBA	2.225	1.71E-12	Extracellular Space	growth factor
KRT20	−2.032	4.90E-12	Cytoplasm	other
MET	1.049	1.87E-07	Plasma Membrane	kinase
MKI67	1.015	1.58E-10	Nucleus	other
MMP2	1.764	2.31E-09	Extracellular Space	peptidase
MUC1	−1.070	1.65E-05	Plasma Membrane	transcription regulator
MUC4	−2.264	3.71E-11	Extracellular Space	growth factor
OSMR	1.166	4.95E-07	Plasma Membrane	transmembrane receptor
PDCD4	−1.572	9.64E-13	Nucleus	other
PDGFRB	1.090	1.47E-10	Plasma Membrane	kinase
PDPN	1.136	5.95E-11	Plasma Membrane	transporter
PTGS2	2.303	5.99E-09	Cytoplasm	enzyme
PTP4A3	1.132	2.47E-12	Plasma Membrane	phosphatase
SERPINE1	1.418	4.29E-09	Extracellular Space	other
SPP1	2.648	6.37E-10	Extracellular Space	cytokine
TIMP1	1.938	9.22E-13	Extracellular Space	other
TNS4	1.486	3.15E-07	Cytoplasm	other
TSPAN8	−1.031	2.83E-09	Plasma Membrane	other

Fold change and p-values were calculated using Integromics biomarker suite.

### Discussion

In this study we attempt to understand the transcription level changes in driver genes affected by SCNAs in colorectal cancer. We queried these genes from three perspectives using gene/exon/network level analysis tools. Our integrated analysis at genomic/transcriptomic levels resulted in finding genes of high priority that can be experimentally studied to establish their role in colorectal cancer. Functional significance of differentially expressed genes confirmed the outcome of our analyses.

Due to the unavailability of high resolution cytogenetic arrays large size of chromosomal regions were implicated in causing colorectal cancer through copy number changes. The commercial SNP genotyping arrays focus on variants that are present in 5% or more of the population and feature a limited number of CNV probes. Therefore, sub microscopic structural variants are poorly captured by available SNP genotyping arrays that were designed to evaluate SNPs. The recent introduction of the Affymetrix CytoScan HD Array (CNV-targeted array), which is based on the validated Genome-Wide Human SNP Array 6.0 and contains more than 2.6 million markers for copy number variants and approximately 750,000 SNPs, has enabled the detection of copy number aberrations with high resolution across the genome [18]. Data from this platform was used to obtain the list of driver genes which could thus be considered to be most precise and accurate. Earlier, results from different groups often lead to a high level of discordance with an overlap of <5% in some cases. GISTIC analysis was able to address this issue and differentiate between passenger and driver mutations with a high level of precision and accuracy [6]. The correlation between expression and CN data is very complex [19] and is very much affected by the type of platform used for generating the data as well as the analysis strategies. Our analysis strategy aimed at reducing the confounding factors. We extracted DNA/RNA from the same piece of tissue in a single protocol [20]. We used matched paired tumour-normal control which is arguably the best way to do a comparative study [21]. Several approaches have been employed for cancer gene prioritization by integrative analysis [22], [23]. We employed a modified two-step approach by filtering the cancer genes using GISTIC and then carried out exon expression analysis.

#### Gene Level

The effect of chromosomal changes is not always direct. Global amplification at genomic level would result into higher level expression of selected genes [9]. Our gene level analysis showed only 20 of 144 driver genes to experience significant change at transcription level. 5 of these genes were listed among the top scoring driver genes. Our results correlate focal amplifications with increased expression levels and have been reported earlier using array CGH (aCGH) for FGFR2, GNAS and AURKA genes [24]. With an average amplicon size of 4.56 Mb, it could be misleading to report all affected genes/non-coding regions to be associated with CRC. FAM46C, EGFR2 and IL6 genes from our analysis have also been listed in the updated cancer gene census [5], [25]. BCAS1 gene that scored the highest in the GISTIC analysis was earlier reported to undergo alternative splicing and downregulation [26] which is consistent with our results. Upregulation of human SLC7A11 mRNA in stromal fibroblast cells from liver metastases is associated with metastatic colorectal cancer in human [27]. PTP4A3 reported to be significantly upregulated in this study has recently been implicated in colon tumorigenesis [28]. ATP8B1 is the gene that has not yet been reported to have any association with colorectal cancer and would be an important molecule for further studies.

Our differential expression analysis of tumour and normal exon array datasets using two independent tools (SAM and LIMMA) showed an overlap of 15 genes with the driver genes. Analysis of>44,000 probes resulting in differentially expressed genes provide an unbiased approach and lends further confidence in the list of overlapping genes. Both LIMMA and SAM approaches generated same results at the functional level as evidenced by Ingenuity Pathway Analysis (IPA).

ABP1, AGR3 and BCAS1 showed downregulation despite amplification at the genome level. This is supported by earlier studies for BCAS1in MCF7 cell line [29]. We observed that the influence of SMAD4 transcription factor was lost in tumour cells and may be responsible for this observation. Similar changes in transcription factors was observed for two other genes indicating the switching behaviour as discussed below.

#### Exon Level

Exon level analysis for measuring gene expression changes is challenging but rewarding. Even the improved algorithms have limitations in providing absolute quantification of the transcript levels. Earlier methods have found exon level data to be more informative about the nature and level of transcripts [30]. Now with the knowledge that more than 90% of all genes undergo alternative splicing to produce more than one transcript for a gene, the potential of exon level data is being realized more than ever [31]–[33]. Gene centric approach for carrying out integrated analysis using aCGH and exon array data has yielded more of confirmatory results and lack the use of full potential of whole genome arrays [34]. FGFR2 gene was shown to be amplified and upregulated which is misleading due to the truncated form of FGFR2. Wt FGFR2 gene was not measured and hence could not be compared with our study [34].

Exon level studies in colorectal cancer have been few and carry several limitations in terms of data analysis. Many of these studies compared tumour and normal from different sources [35]. Our results show a subset of differentially expressed genes to experience change in splicing pattern. 29 exons belonging to 13 genes showed significant splice index (SI) and MiDAS p-values. Both the values are strong indicators to measure alternative splicing. MUC4 is very well known to undergo alternative splicing and cause cancer [36] but the alternative splicing of IL6 is novel in association with CRC. ADAM12 that scored a high SI value has been implicated earlier in lung cancer [37]. 5 genes (ACTN1, CALD1, SLC3A2, CTTN and FN1) reported earlier as differentially spliced [38] have been found in the our study as well which used a different analysis strategy.

ANK3 is known to use alternative transcription start sites in colorectal cancer [8] and could also be the mechanism for other gene MYLK for which we did not see a significant change in gene level expression.

#### Network Level

Pathways analysis has been used to measure the relevance of the genes affected by CNAs by creating networks among them [1]. However, these networks are limited in their useful interpretation owing to the absence of directionality. Our causal network analysis provides more useful information on the genes involved in these networks. We observed a significant difference in the number of target genes between tumour and normal. In case of genes found in the amplified region but were downregulated we observed a loss of the transcription factor regulation, whereas in the upregulated gene found in the deleted region there was a switch in the transcription factor. From the list of driver genes we chose the transcription factors and studied their change in behaviour in tumour samples. AHR has been established as a tumour suppressor gene in colon and other cancers [39]. This study further explains the enhanced role of AHR by the increased number of outbound (target) genes in tumour. Our study provides evidence that TSHZ1 loses its role as master regulator in normal cells while RUVBL1 assumes that role. These provide interesting opportunities for mechanistic studies of network/pathways affected in CRC. It has been envisaged through integrated studies that many different genomic alterations potentially dys-regulate the same pathways in complex diseases [40]. Further studies of the regulatory level changes in this study will be able to establish this concept in CRC.

Functional role of the differentially expressed genes and the identification of MYC, MMP and IL6 as important nodes in the affected networks provide leads that need to be validated to establish their association with CRC. Biomarkers, especially MUC4 will be an important molecule to study mechanistically and establish their use in clinical studies. This study provides wealth of analyzed data and an enriched list of genes that can serve as potential clues to understand the biology of colorectal cancer.

### Supporting Information

Figure S1 **Significance of Microarray analysis.** SAM analysis was performed using Integromics biomarker discovery suites on all samples. The results were complimentary to LIMMA analysis as reflected in the number of differentially expressed genes.
(TIF)

Figure S2 **Splice index and exon expression plots for remaining 11 genes.** Splice index and exon expression values of all 13 genes that were found significant among the driver genes were plotted. Comparison of ‘Normal’ and ‘Tumor’ samples is depicted to observe the change in splice index as well the expression pattern at exon level.
(DOCX)

Figure S3 Biological processes enrichment chart for differentially expressed genes. This bar plot shows the differentially expressed genes (as obtained from Integromics) are enriched in three functions viz., cell division, mitosis and cell adhesion.
(PNG)

Table S1 **Types and stages of all the patient samples used in the study.**
(DOCX)

Table S2 **Fold change values of all 144 GISTIC genes as calculated by AltAnalyze Program.**
(TXT)
