# Correction: Integrated Exon Level Expression Analysis of Driver Genes Explain Their Role in Colorectal Cancer

**DOI:** 10.1371/journal.pone.0122532

**Published:** 2015-03-26

**Authors:** The PLOS ONE Staff

The affiliations for the sixth and seventh authors are incorrect. The affiliation for AbdulAziz AlFahed is: Salman Bin Abdulaziz University, Al kharj, Saudi Arabia. The affiliation for Glowi Alasiri is: Al-Imam Muhammad Ibn Saud Islamic University, Riyadh, Saudi Arabia.
