# Supplementary figures and images for: Integrated Exon Level Expression Analysis of Driver Genes Explain Their Role in Colorectal Cancer
Source: PLoS One. 2014 Oct 21;9(10):e110134. doi: 10.1371/journal.pone.0110134 (PMC4204855; doi:10.1371/journal.pone.0110134)

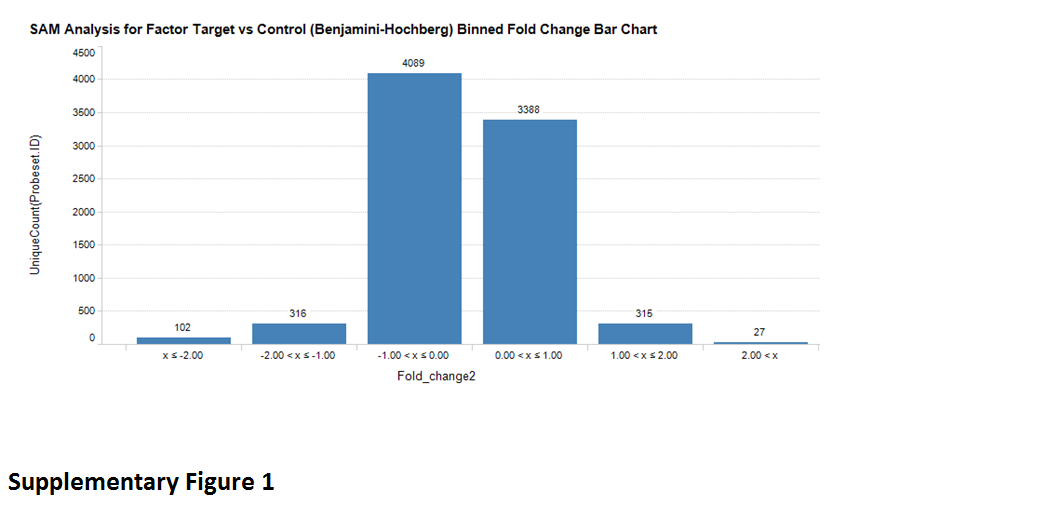

Supplement: Figure S1 — Significance of Microarray analysis. SAM analysis was performed using Integromics biomarker discovery suites on all samples. The results were complimentary to LIMMA analysis as reflected in the number of differentially expressed genes. (TIF) [file pone.0110134.s001.tif]

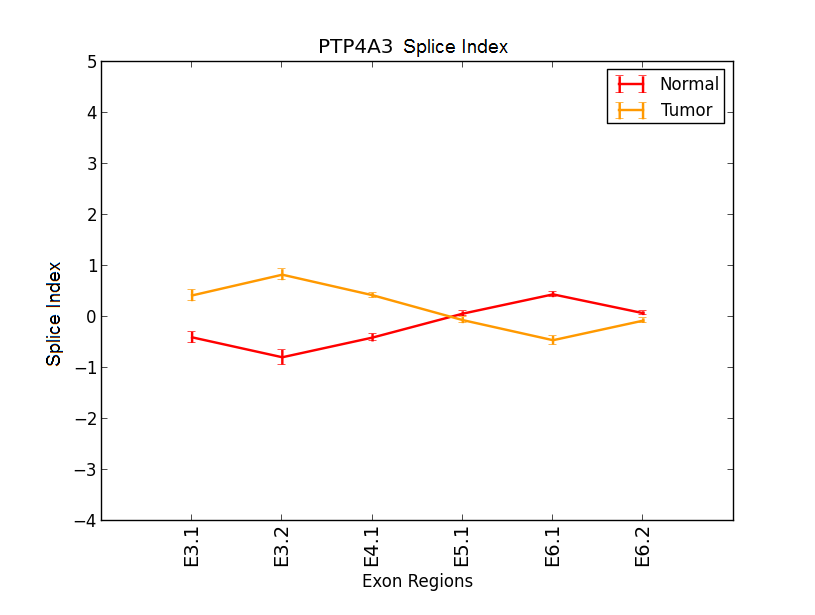

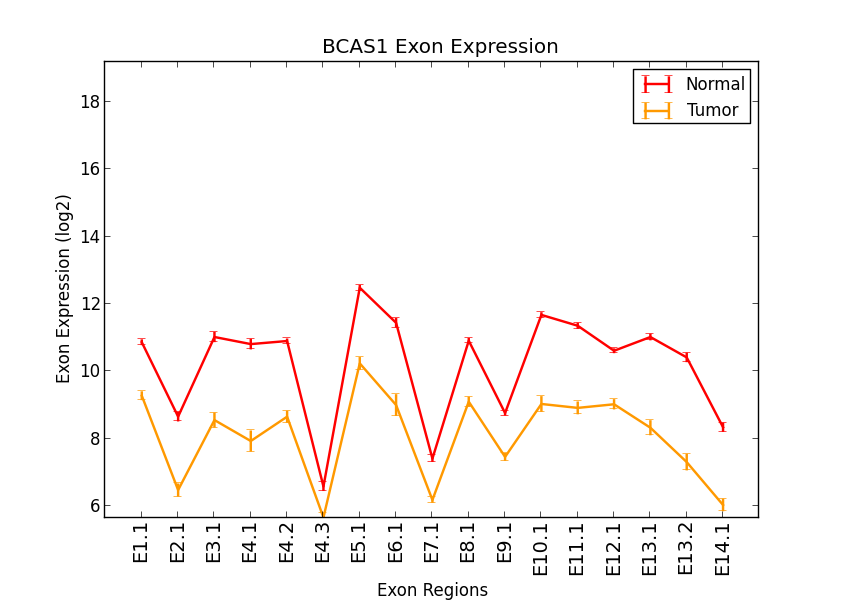

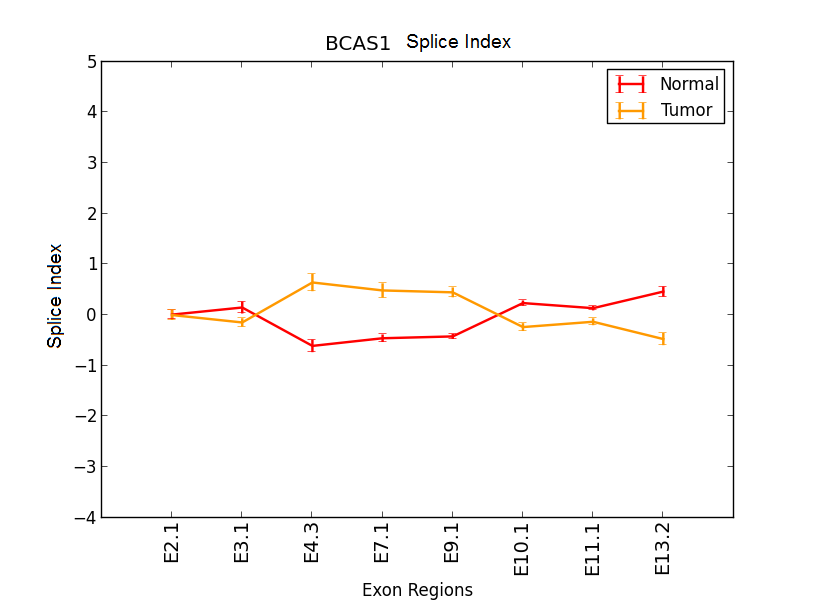

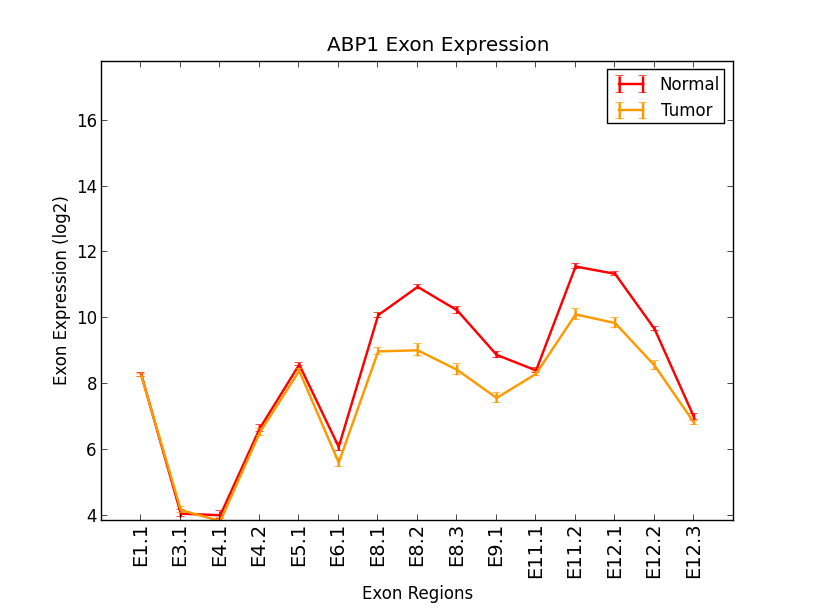

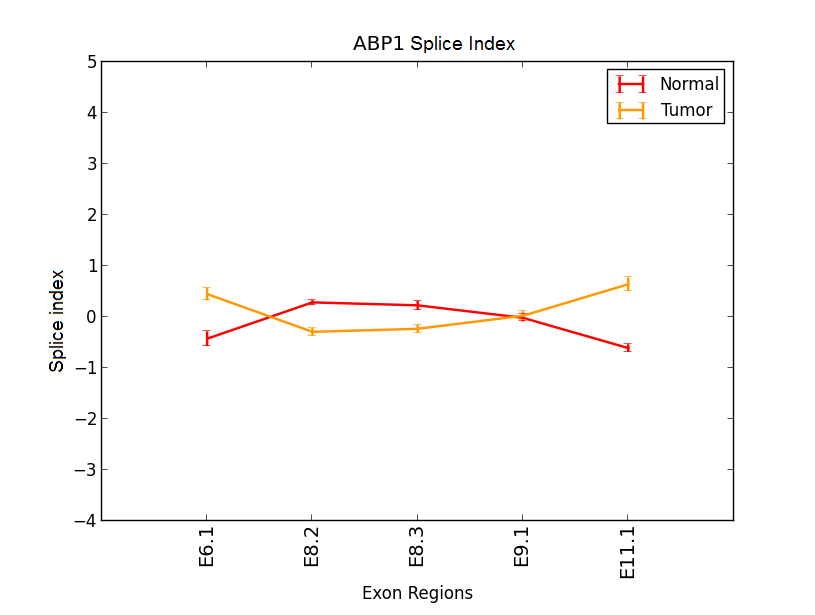


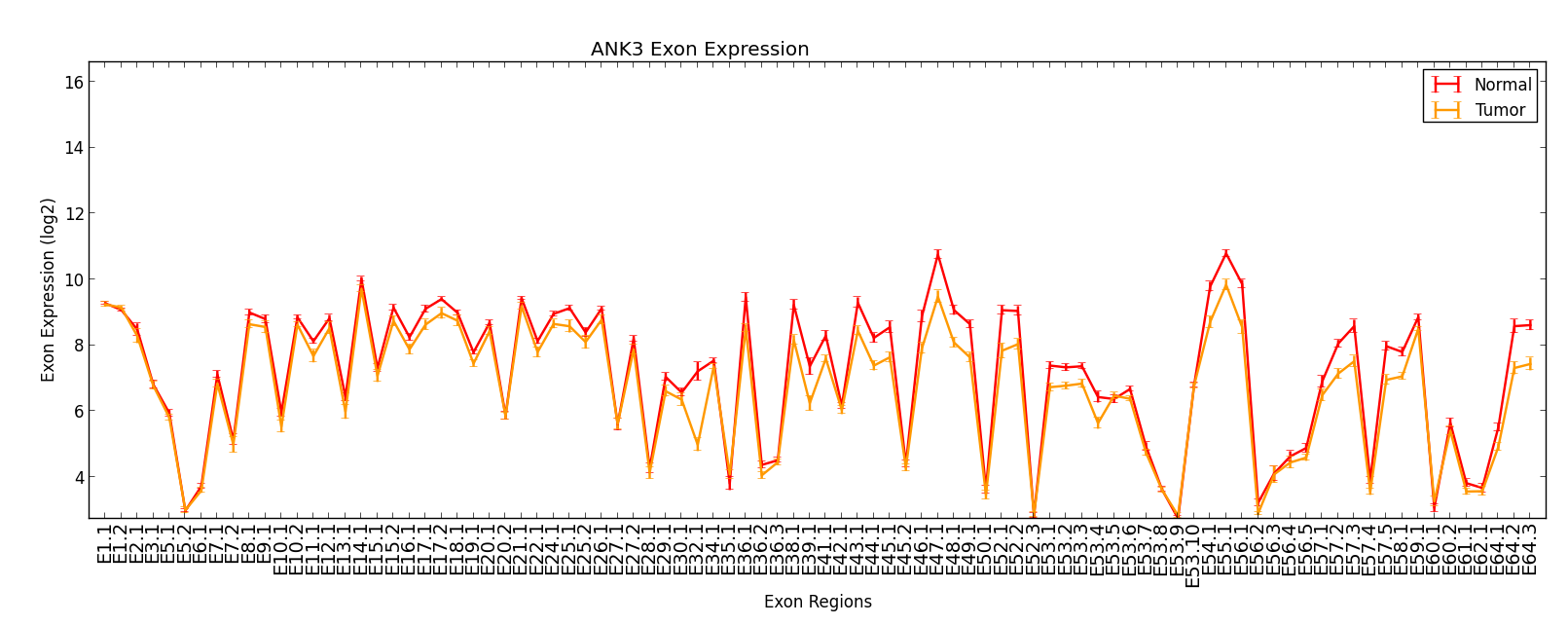

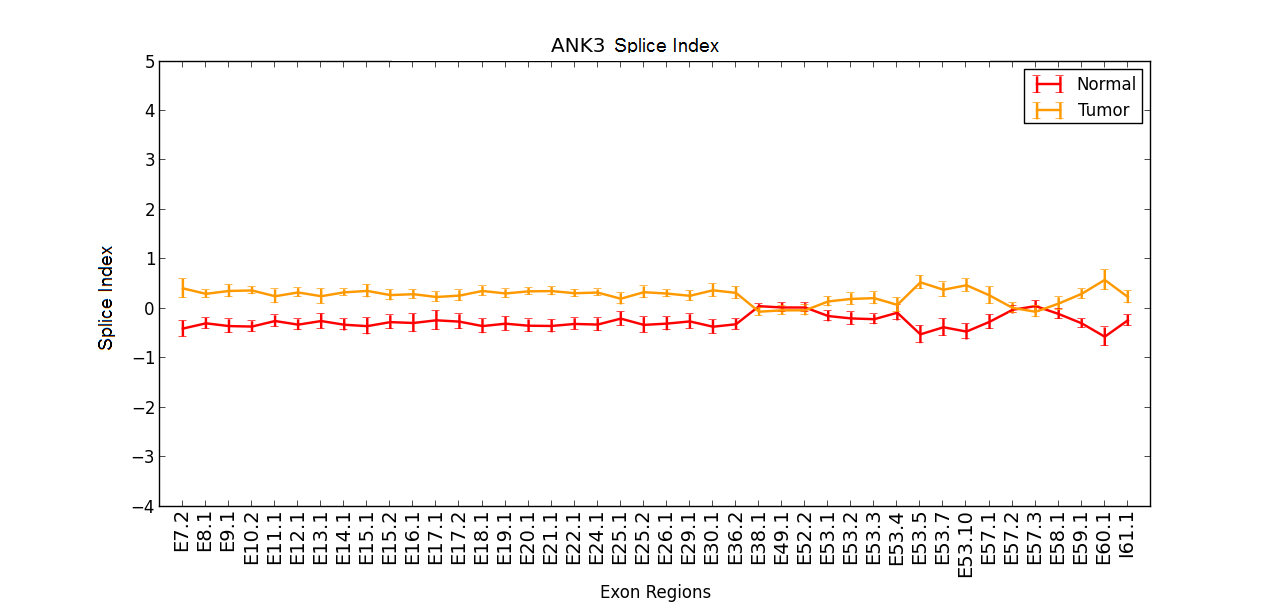


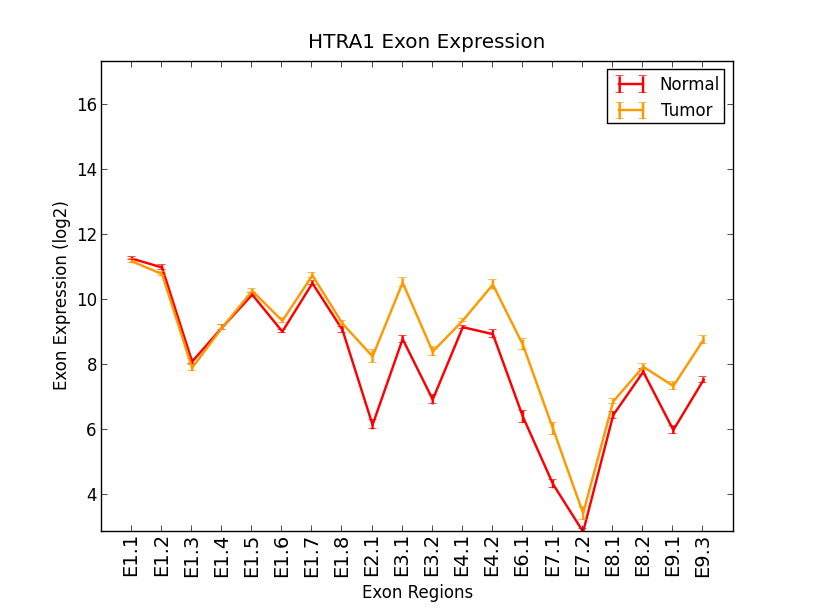

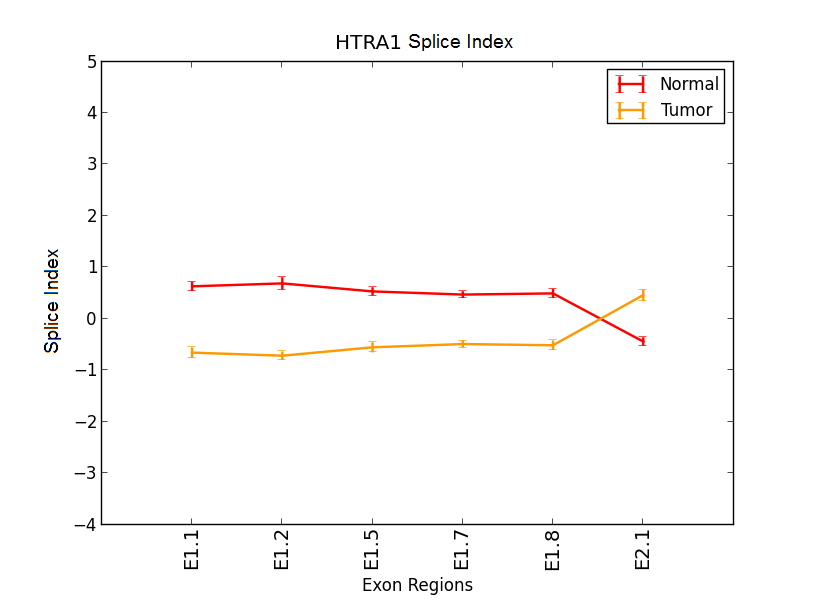


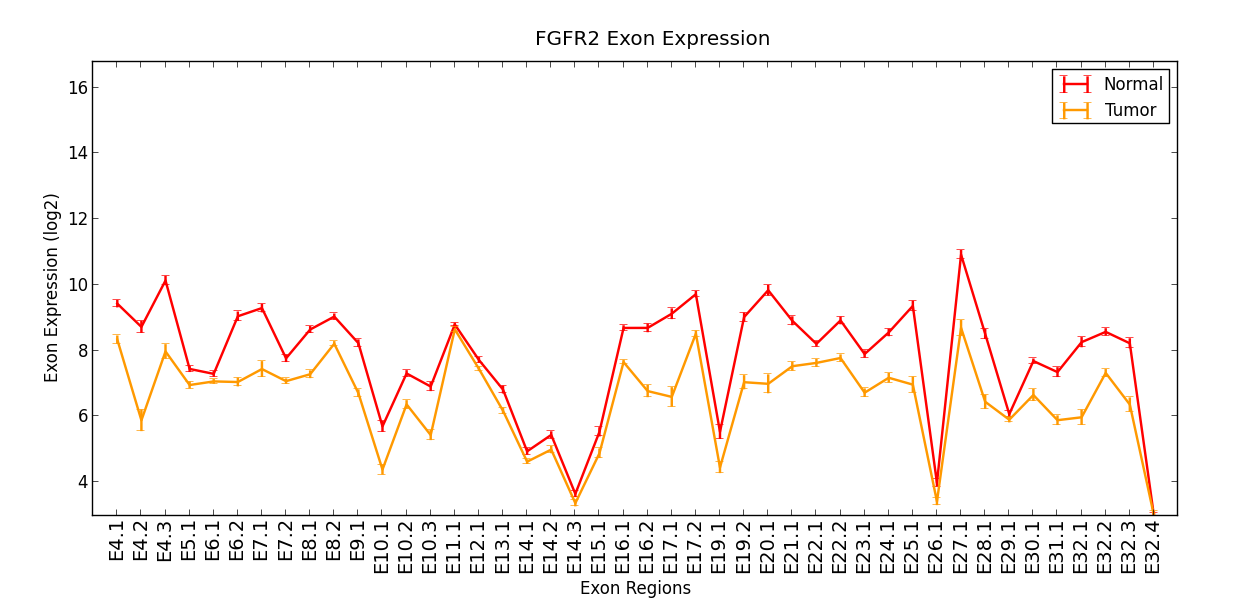

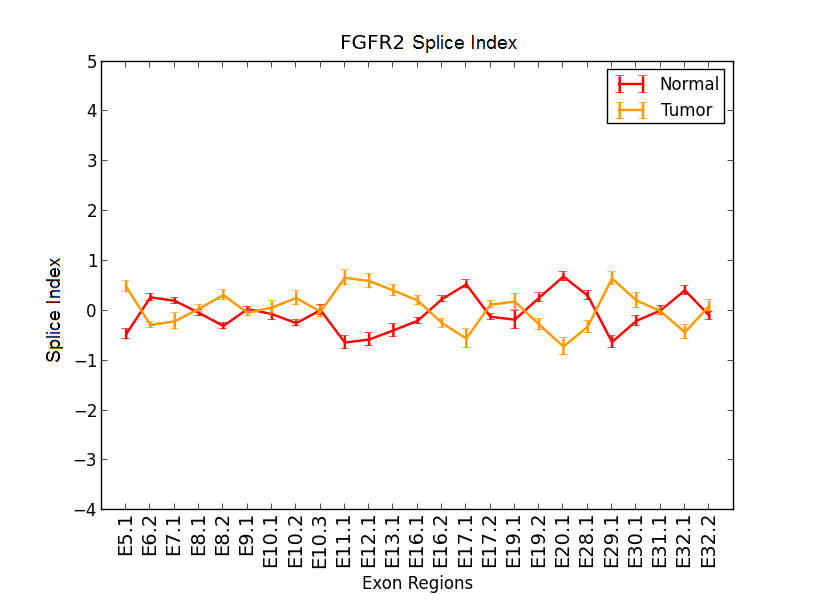


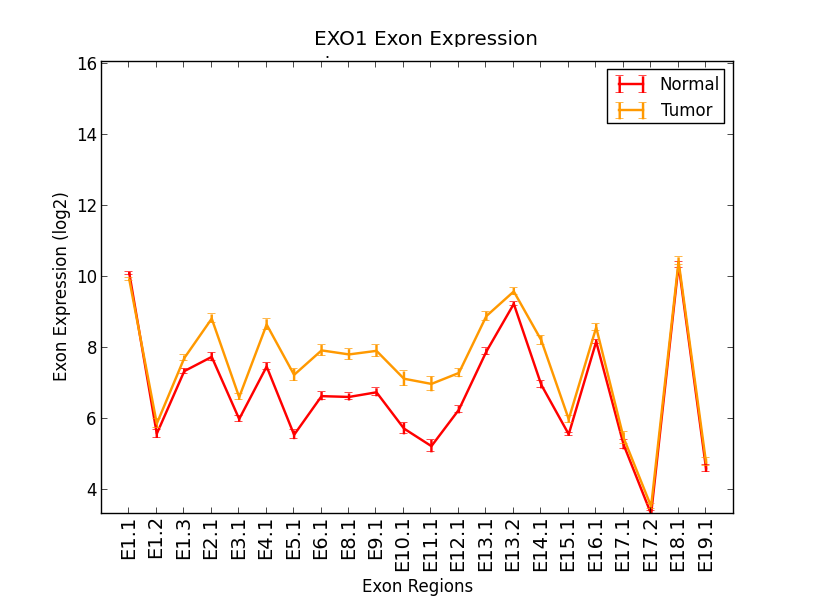

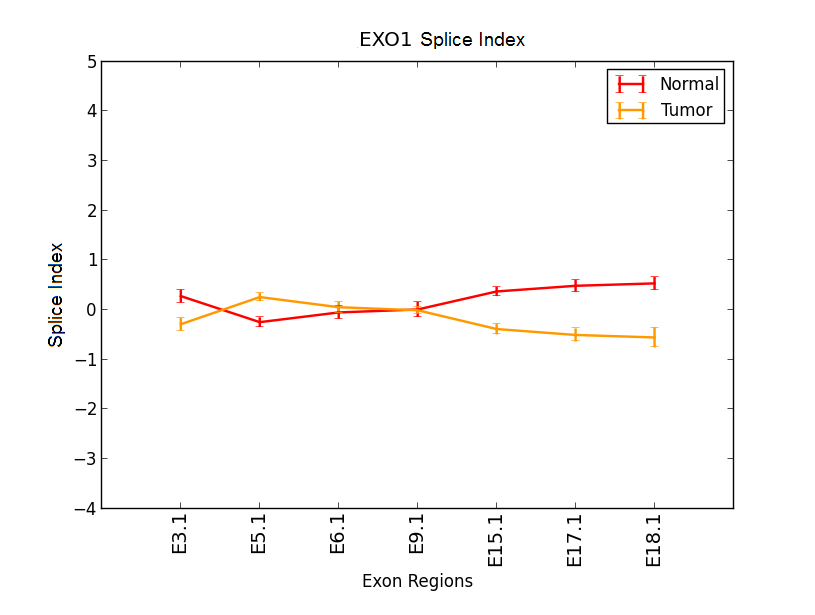

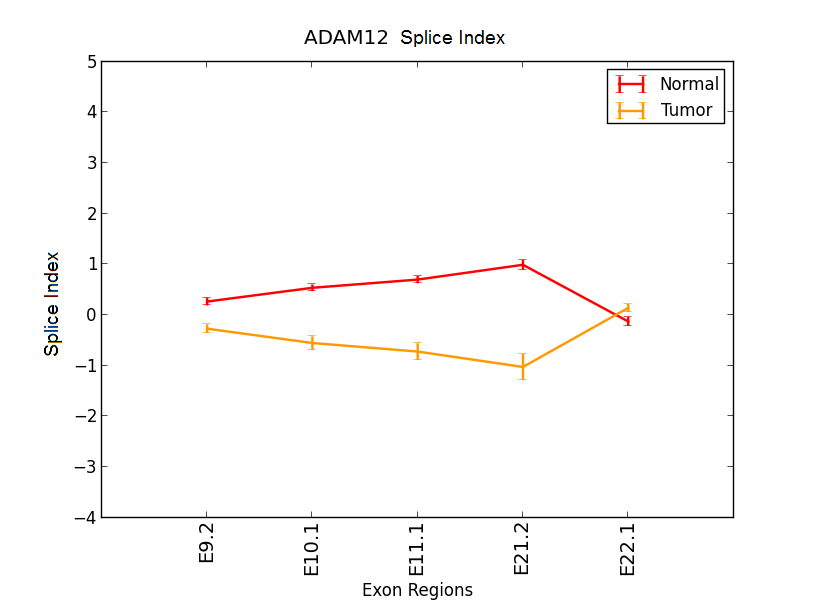

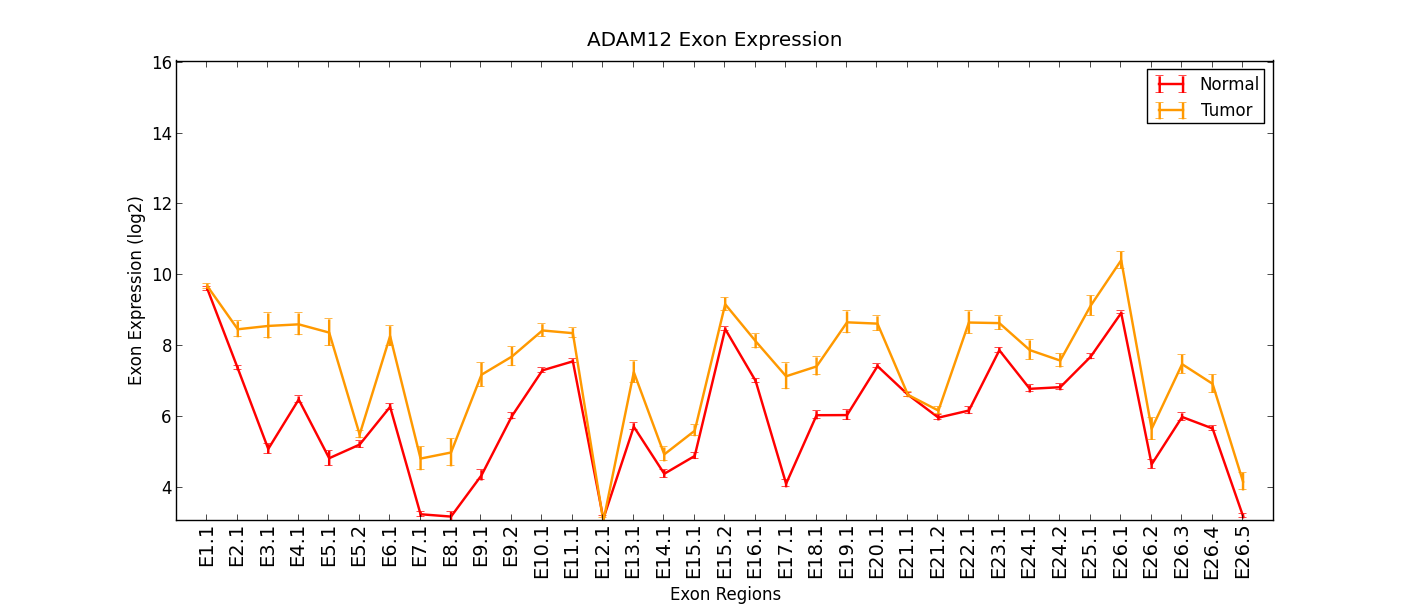


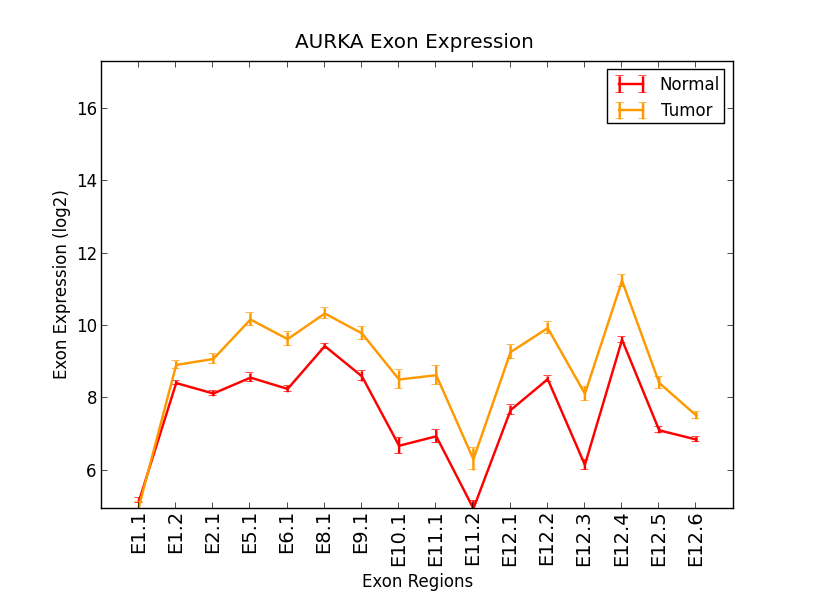

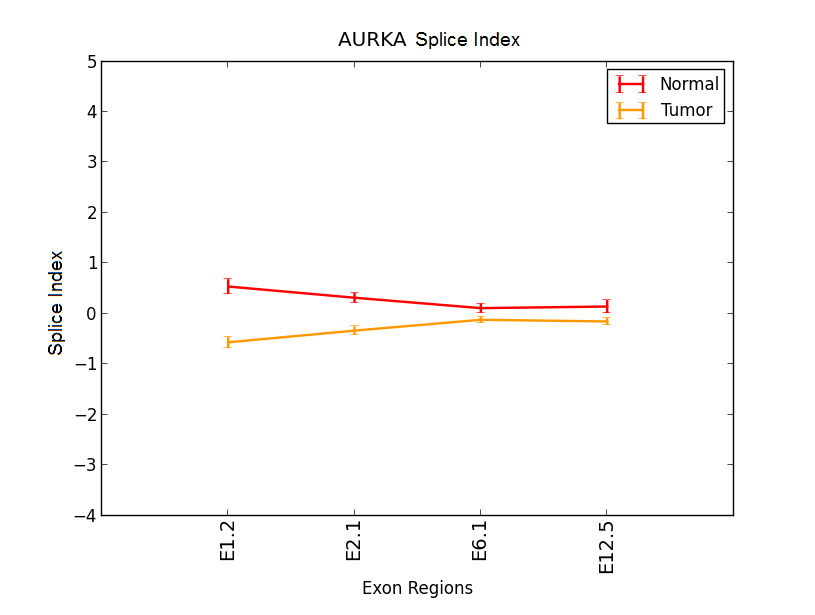


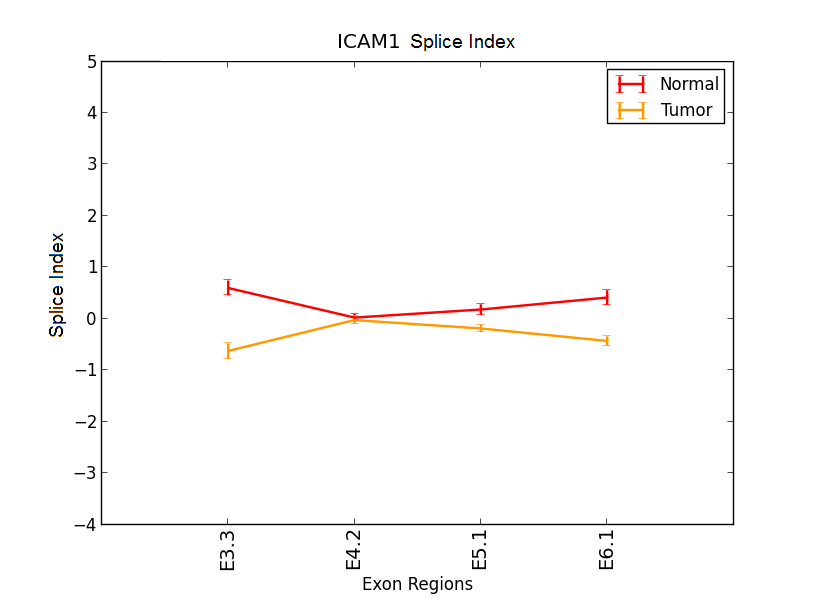

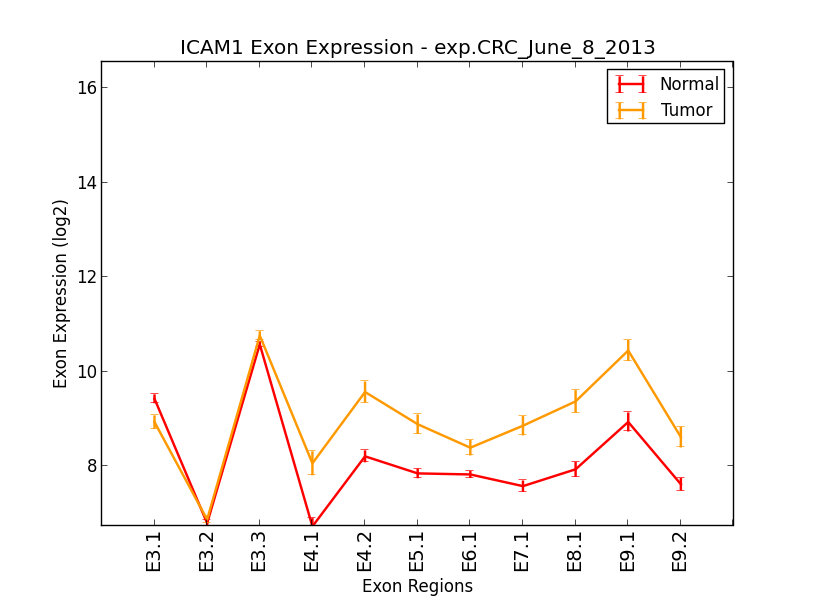


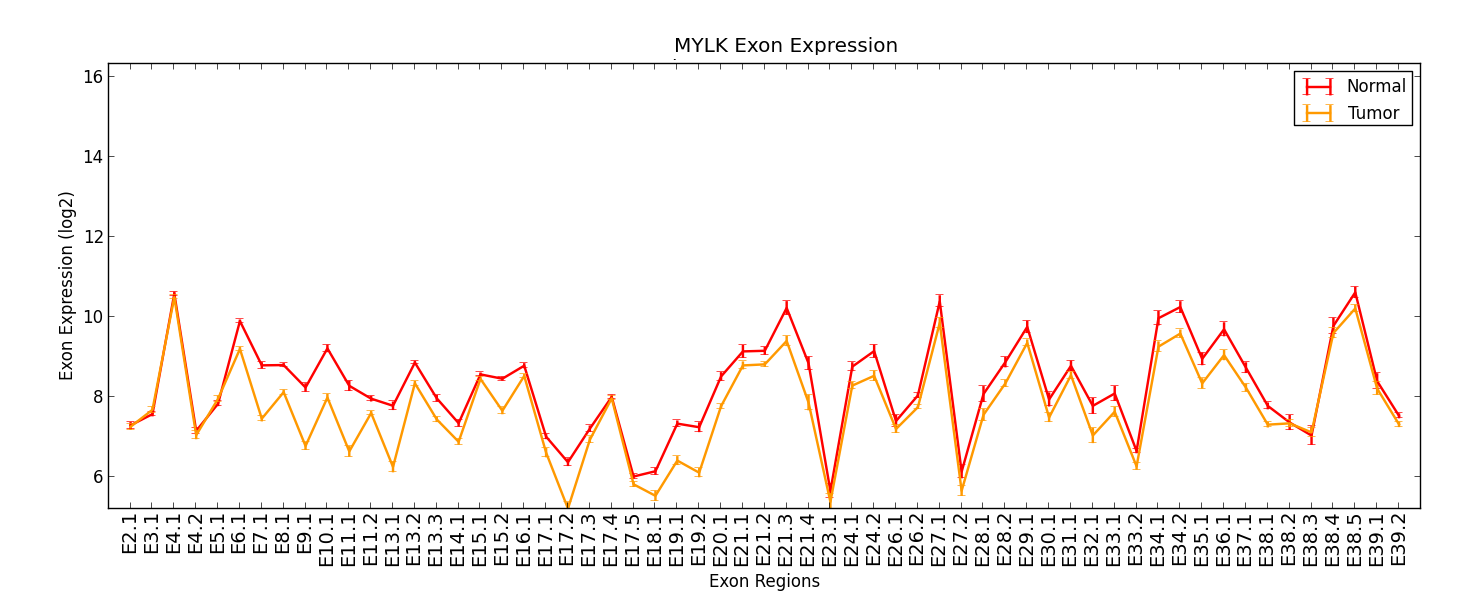

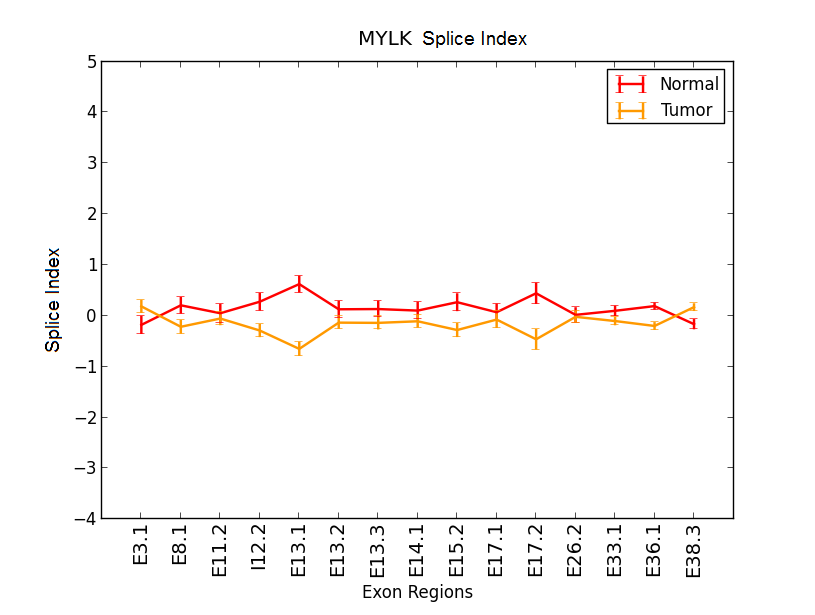

Supplement: Figure S2 — Splice index and exon expression plots for remaining 11 genes. Splice index and exon expression values of all 13 genes that were found significant among the driver genes were plotted. Comparison of ‘Normal’ and ‘Tumor’ samples is depicted to observe the change in splice index as well the expression pattern at exon level. (DOCX) [file pone.0110134.s002.docx]

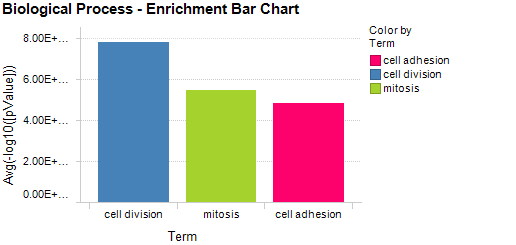

Supplement: Figure S3 — Biological processes enrichment chart for differentially expressed genes. This bar plot shows the differentially expressed genes (as obtained from Integromics) are enriched in three functions viz., cell division, mitosis and cell adhesion. (PNG) [file pone.0110134.s003.png]
