# Supplementary material for: Integrated Exon Level Expression Analysis of Driver Genes Explain Their Role in Colorectal Cancer
Source: PLoS One. 2014 Oct 21;9(10):e110134. doi: 10.1371/journal.pone.0110134 (PMC4204855; doi:10.1371/journal.pone.0110134)
Supplement: Table S1 — Types and stages of all the patient samples used in the study. (DOCX) [file pone.0110134.s004.docx]

Table S1: Types and stages of all the patient samples used in the study

| Sample ID | Type of tumor | Stage of tumor |
| --- | --- | --- |
| 050911-01-T/N-S | Mucinous adenocarcinoma, moderately differentiated | T3N0M1 |
| 051611-01-T/N-B | adenocarcinoma, moderately differentiated | T2N0M0 |
| 052811-01-T/N-B | adenocarcinoma, moderately differentiated | T3N0M0 |
| 062011-01-T/N-B | adenocarcinoma, moderately differentiated | T2N0M0 |
| 070911-01-T/N-B | adenocarcinoma, moderately differentiated | T3N0M0 |
| 072511-01-T/N-S | adenocarcinoma, moderately differentiated | T3N1M0 |
| 072711-01-T/N-S | adenocarcinoma, poorly differentiated | T3N1M1 |
| 073011-01-T/N-S | adenocarcinoma, well differentiated | T3N0M0 |
| 092511-01-T/N-B | adenocarcinoma, moderately differentiated | T3N0M0 |
| 092611-01-T/N-B | adenocarcinoma, moderately differentiated | T3N2M1 |
| 092811-01-T/N-B | adenocarcinoma, moderately differentiated | T1N0M0 |
| 101011-01-T/N-B | adenocarcinoma, moderately differentiated | T3N1M1 |
| 112611-03-T/N-B | adenocarcinoma, moderately differentiated | T3N1M0 |
| 112911-01-N/T-S | Mucinous adenocarcinoma, moderately differentiated | T3N2M1 |
| 120511-01-T/N-S | adenocarcinoma, moderately differentiated | T3N1M1 |
| 121011-01-T/N-B | adenocarcinoma, moderately differentiated | T3N2M0 |
| 120112-01-T/N-B | adenocarcinoma, moderately differentiated | T3N0M1 |
| 010912-01-T/N-B | adenocarcinoma, moderately differentiated | T3N0M0 |
| 032612-01-T/N-S | adenocarcinoma, moderately differentiated | T3N0M0 |
| 033112001-T/N-B | adenocarcinoma, moderately differentiated | T3N0M0 |
| 042412-01-T/N-B | adenocarcinoma, moderately differentiated | T2N1M0 |
| 060612-01-T/N-S | adenocarcinoma, moderately differentiated | T3N0M0 |
| 101512-01-T/N-S | adenocarcinoma, moderately differentiated | T3N0M0 |
| 010213-01-T/N-B | adenocarcinoma, moderately differentiated | T3N2M0 |
| 030613-01-T/N-B | adenocarcinoma, moderately differentiated | T3N0M1 |
| 061013-01-T/N-S | adenocarcinoma, moderately differentiated | T3N0M0 |
| 070113-01-T/N-S | adenocarcinoma, moderately differentiated | T3N1M1 |
| 121613-01-T/N-S | adenocarcinoma, moderately differentiated | T2N0M0 |
| 010714-01-T/N-S | adenocarcinoma, moderately differentiated | T3N1M0 |
| 012214-01-T/N-S | adenocarcinoma, moderately differentiated | T3N0M0 |
| 033114-01-T/N-S | adenocarcinoma, moderately differentiated | T3N0M0 |
| 042214-01-T/N-S | adenocarcinoma, moderately differentiated | T2N1M0 |
